# Deep Sequencing the microRNA profile in rhabdomyosarcoma reveals down-regulation of miR-378 family members

**DOI:** 10.1186/1471-2407-14-880

**Published:** 2014-11-25

**Authors:** Francesca Megiorni, Samantha Cialfi, Heather P McDowell, Armando Felsani, Simona Camero, Alessandro Guffanti, Barry Pizer, Anna Clerico, Alessandra De Grazia, Antonio Pizzuti, Anna Moles, Carlo Dominici

**Affiliations:** Department of Paediatrics and Infantile Neuropsychiatry, Sapienza University of Rome, Viale Regina Elena 324, 00161 Rome, Italy; Department of Molecular Medicine, Sapienza University of Rome, Viale Regina Elena 324, 00161 Rome, Italy; Department of Oncology, Alder Hey Children’s NHS Foundation Trust, Eaton Road, L12 2AP Liverpool, United Kingdom; School of Reproductive and Developmental Medicine, University of Liverpool, L12 2AP Liverpool, United Kingdom; Genomnia srl, Via Nerviano 31/b, 20020 Lainate, MI Italy; Department of Experimental Medicine, Sapienza University of Rome, Viale Regina Elena 324, 00161 Rome, Italy

**Keywords:** Rhabdomyosarcoma, MicroRNAs, Deep sequencing, miR-378a-3p, 5-aza-2′-deoxycytidine

## Abstract

**Background:**

Rhabdomyosarcoma (RMS) is a highly malignant tumour accounting for nearly half of soft tissue sarcomas in children. MicroRNAs (miRNAs) represent a class of short, non-coding, regulatory RNAs which play a critical role in different cellular processes. Altered miRNA levels have been reported in human cancers, including RMS.

**Methods:**

Using deep sequencing technology, a total of 685 miRNAs were investigated in a group of alveolar RMSs (ARMSs), embryonal RMSs (ERMSs) as well as in normal skeletal muscle (NSM). Q-PCR, MTT, cytofluorimetry, migration assay, western blot and immunofluorescence experiments were carried out to determine the role of miR-378a-3p in cancer cell growth, apoptosis, migration and differentiation. Bioinformatics pipelines were used for miRNA target prediction and clustering analysis.

**Results:**

Ninety-seven miRNAs were significantly deregulated in ARMS and ERMS when compared to NSM. MiR-378 family members were dramatically decreased in RMS tumour tissue and cell lines. Interestingly, members of the miR-378 family presented as a possible target the insulin-like growth factor receptor 1 (IGF1R), a key signalling molecule in RMS. MiR-378a-3p over-expression in an RMS-derived cell line suppressed IGF1R expression and affected phosphorylated-Akt protein levels. Ectopic expression of miR-378a-3p caused significant changes in apoptosis, cell migration, cytoskeleton organization as well as a modulation of the muscular markers MyoD1, MyoR, desmin and MyHC. In addition, DNA demethylation by 5-aza-2′-deoxycytidine (5-aza-dC) was able to up-regulate miR-378a-3p levels with a concomitant induction of apoptosis, decrease in cell viability and cell cycle arrest in G2-phase. Cells treated with 5-aza-dC clearly changed their morphology and expressed moderate levels of MyHC.

**Conclusions:**

MiR-378a-3p may function as a tumour suppressor in RMS and the restoration of its expression would be of therapeutic benefit in RMS. Furthermore, the role of epigenetic modifications in RMS deserves further investigations.

**Electronic supplementary material:**

The online version of this article (doi:10.1186/1471-2407-14-880) contains supplementary material, which is available to authorized users.

## Background

Rhabdomyosarcoma (RMS) is the most common soft tissue sarcoma in childhood [1], representing approximately 50% of all sarcomas in children aged 0–14 years and 4-5% of malignant solid tumours in the paediatric population. The two major histological subtypes, alveolar rhabdomyosarcoma (ARMS) and embryonal rhabdomyosarcoma (ERMS), have distinct clinical features and outcomes. ERMSs are more frequent (~80% of cases) and generally affect younger children (0–4 years); they occur more commonly in the neck, head and genito-urinary tract [2]. As the name implies, tumour cells resemble embryonal skeletal muscle cells. ARMSs (~20% of cases) usually present throughout childhood, typically originating in the limbs and trunk, often with regional or metastatic lymph node involvement already at diagnosis, and carry a significantly worse outcome [2, 3]. ARMS is so called because tumour cells form small spaces or pseudo-alveoli.

The role of genetic factors in the development of RMS has been confirmed by several recent epidemiological observations and advances in molecular genetics. Although the majority of RMS cases are sporadic, *i.e.* not associated with hereditary syndromes, a small proportion are linked to congenital anomalies, *e.g.* Beckwith-Wiedemann syndrome, or are associated with particular familial syndromes, such as neurofibromatosis type I and Li-Fraumeni syndrome [4–6]. ARMS and ERMS are both characterised by particular genetic alterations that are likely to play a decisive role in cancer pathogenesis. Eighty percent of ARMS tumours have either t(2;13)(q35,q14) or t(1;13)(p36;q14) chromosomal translocations, which generate PAX3-FOXO1 and PAX7-FOXO1 fusion proteins, respectively [7]. However, constitutive expression of PAX3/7-FOXO1 chimeric genes is not sufficient to induce RMS development in transgenic animals [8, 9]. Loss of heterozygosity of the short arm of chromosome 11 (11p15.5), with over-expression of the insulin-like growth factor II, is often associated with ERMS [10]. However, although several tumour causative genes have been identified, a detailed understanding of the molecular mechanisms underlying RMS development has not yet been achieved.

Recent studies have suggested that microRNAs (miRNAs) may play an essential role in RMS [11, 12]. MiRNAs are a class of conserved, short, non-coding molecules which regulate gene expression through binding to non-perfect complementary sequences at the 3′-untranslated regions (UTRs) of target messenger RNAs (mRNAs), resulting in translational repression [13]. Several pre-miRNAs stem-loops are processed to produce two mature and functional miRNAs, designated with the suffix “-3p” or “-5p,” depending on the originating hairpin arm [14]. It has been predicted that about one-third of all mammalian genes are targeted by miRNAs [15, 16]. Deregulation of miRNA expression is associated with various cancers and many studies indicate that miRNAs may act either as oncogenes or tumour suppressors [17–19], controlling key processes in tumorigenesis, such as tumour initiation, progression and metastatic spread. An increasing number of miRNAs, such as miR-1, miR-133a, miR-200c, miR-206, miR-214 and miR-9*, have been identified to have a role in RMS [11, 20–24], as recently summarized by Novak et al. [12].

Epigenetic DNA changes, such as DNA promoter hypermethylation and histone modifications, have critical roles in chromatin remodelling and general regulation of gene expression in mammalian development and human diseases [25]. In particular, DNA methylation of CpG islands in promoter regions has been correlated with silencing of tumour suppressor genes and other tumour-related genes, and it has been recognised as a crucial component of the mechanism underlying cancer development [26]. DNA methylation-associated silencing of miRNAs in different human tumours, including RMS, has also been reported [27, 28].

In this study, deep sequencing technology was utilised to quantify the absolute abundance of miRNAs in ARMS and ERMS tumours as well as in normal skeletal muscle (NSM), and to identify an RMS-specific miRNA expression pattern. The majority of miRNAs were found to be down-regulated, as predicted. Interestingly, miR-378a/b/c/d/e/f/h/i molecules, belonging to a large family of evolutionary conserved miRNAs, were strongly under-represented in ARMS and ERMS tumours in comparison to NSM. Transient transfection of miR-378a-3p in an ARMS-derived cell line (RH30) induced apoptosis and decreased viability/proliferation by repressing the IGF1R/AKT pathway. Importantly, elevated levels of miR-378a-3p impaired RH30 cell migration and promoted myogenic differentiation. Demonstration that epigenetic modifications may be involved in RMS tumourigenesis was achieved by restoring miR-378a-3p levels. In addition, 5-aza-2′-deoxycytidine (5-aza-dC) treatment induced apoptosis, cell cycle arrest in G2 phase and decreased cell viability compared to untreated RH30 cells. Interestingly, RH30 5-aza-dC-treated cells changed their morphology and expressed muscle differentiation markers, partially overlapping the effect of miR-378a-3p transfection.

Taken together, these data provide the first evidence for an anti-tumour activity of miR-378a-3p in RMS, suggesting that this miRNA could be a potential therapeutic target in RMS. Furthermore, the importance of epigenetic regulation in RMS was confirmed, which may have important clinical implications in this malignancy.

## Methods

### Patient clinical and tumour histopathological characteristics

Fifteen RMS tumour samples, 7 ARMSs and 8 ERMSs, were obtained at diagnosis before any treatment from children admitted to the Department of Paediatrics and Infantile Neuropsychiatry at “Sapienza” University, and to the Department of Oncology at Alder Hey Children’s NHS Trust, Liverpool. Histopathological diagnosis was confirmed using immunohistochemistry. All 7 ARMS were investigated for PAX3/7-FOXO1 translocations using standard FISH analysis: 5 tumours were PAX3-FOXO1–positive, 1 was PAX7-FOXO1–positive and 1 was fusion-negative. Patients were grouped according to the Intergroup Rhabdomyosarcoma Study (IRS) postsurgical grouping system [29]. Details of the patients are described in Table 1. ARMS1-2-3-4 and ERMS1-2-3-4 tumour samples were used for deep sequencing study. Institutional written informed consent was obtained from the patient’s parents or legal guardians. The study underwent ethical review and approval according to the local institutional guidelines (Policlinico Umberto I’s Ethics Committee and Alder Hey Children’s NHS Foundation Trust Ethics Committee). Control RNA was extracted from normal skeletal muscle (NSM) obtained from eight children undergoing surgery for benign conditions.

**Table 1:** **Clinico-pathological features of the analysed tumour cases**

Case	Histology	Fusion status	Primary site	Clinical stage
ARMS1*	alveolar	PAX3	trunk	III
ARMS2*	alveolar	PAX3	extremity	III
ARMS3*	alveolar	negative	trunk	II
ARMS4*	alveolar	PAX7	uterus-vagina	III
**ARMS7**	alveolar	PAX3	extremity	IV
**ARMS36**	alveolar	PAX3	trunk	III
**ARMS37**	alveolar	PAX3	uterus-vagina	II
ERMS1*	embryonal	n.a.	retroperitoneum	III
ERMS2*	embryonal	n.a.	bladder-prostate	III
ERMS3*	embryonal	n.a.	bladder-prostate	III
ERMS4*	embryonal	n.a.	trunk	III
**ERMS12**	embryonal	n.a.	bladder-prostate	III
**ERMS21**	embryonal	n.a.	retroperitoneum	III
**ERMS23**	embryonal	n.a.	uterus-vagina	II
**ERMS27**	embryonal	n.a.	extremity	I

Variables were categorized as follows: histological subtype, embryonal *versus* alveolar; gene fusion status; primary site and clinical stage. Fusion status PAX3: PAX3-FOXO1–positive; PAX7: PAX7-FOXO1–positive ; n.a. - not applicable. The symbol * indicates samples used for deep sequencing analysis.

### RNA isolation

Samples were immediately frozen in liquid nitrogen after surgery and stored at −80°C. Total RNA was extracted using TRIzol (Invitrogen) according to the manufacturer’s instructions. Samples were enriched for small RNAs up to 200 bp by size selection using Pure Link miRNA Isolation Kit (LifeTech). RNA purity, integrity and size distribution were assessed using an Agilent 2100 Bioanalyzer (Agilent Technologies).

### Small RNA library generation and sequencing

Enriched RNA samples were processed using the Small RNA Expression Kit according to the manufacturer’s protocol (Small RNA expression kit, rev. C, Applied Biosystems). Briefly, RNA was first hybridized and ligated with the adapter mix “A”, subsequently reverse transcribed and treated with RNAse H. The obtained cDNA libraries were PCR amplified, purified and size-selected by PAGE, resulting in libraries containing inserted small RNA sequences of 20–40 bp length. Size, integrity and purity of the libraries were verified by the Agilent 2100 Bioanalyzer (Agilent Technologies). cDNA libraries were barcoded using the Solid RNA barcoding kit and amplified onto beads using emulsion PCR. Templated beads were deposited on slides and analysed using the Applied Biosystems SOLiD 4 Sequencer.

### Statistical and bioinformatics analyses

The quality filtered reads were mapped against all annotated human mature miRNA sequences (miRBase v19.0) [30] using the Lifetech Lifescope 2.5.1 Small RNA pipeline, filtering for rRNA, primers, and small non-miRNA non-coding transcribed sequences such as tRNAs and snoRNAs. Sequence counts were extracted and reformatted with perl scripts from the pipeline output. Differential expression analysis was performed with the edgeR Bioconductor statistical library [31] version 3.0.8 on R version 2.15.3. TMM-normalized sequence counts in the libraries were transformed in Counts Per Millions (CPM) according to the formula: CPM = (normalized counts/total miRNA matches) *1,000,000. After having estimated the tagwise dispersion, genewise exact test [32] as implemented in edgeR was used to measure the significance of differential expression, using the gene “Pseudo-counts”. Sequences were deemed significantly differentially expressed if (1) the p-value given by this method was < 0.05, (2) the total count was greater than 50 CPM in at least one group of samples, and (3) there was at least a two-fold change in normalised sequence counts between the two groups.

The validated target prediction of a panel of regulated microRNAs was performed by interrogating the ‘Validated Target’ option of the miRWalk web software [33]. Target genes of the miR-378 family were predicted using TargetScan Human 6.2 (http://www.targetscan.org/), miRanda (http://www.microrna.org/microrna/home.do) and DIANA-microT version 3.0 (http://diana.cslab.ece.ntua.gr/microT/) algorithms. Annotation and enrichment of functional pathways associated with the miR-378 target genes were evaluated using the Reactome database and associated analytical tools (http://www.reactome.org/). The same miR-378 target gene list was used as the starting dataset for the generation of a Functional Interaction network analysis and related Gene Ontology enrichment analysis as described [34, 35]. Preliminary isoMIR analysis was performed by selecting from the YM500 miR-Seq database (http://ngs.ym.edu.tw/ym500v2/index.php) the hsa-miR378a-3p isoforms which had been found with at least 50.000 sequence reads in 40 different experiments, allowing 3 nt 5′ extensions, 3′ extensions and one mismatch with respect to the reference miRBase mature sequence. The resulting 14 isomirs were identified and counted in all the NSM, ARMS and ERMS sequences using the bowtie 0.12.8 database search software and ad-hoc created perl scripts.

Nucleotide sequence pattern analysis of the miR-378a-3p promoter region was performed using the program CpG plot of the EMBOSS sequence analysis suite (http://emboss.sourceforge.net/), with standard parameters, and other softwares such as CpG island searcher (http://cpgislands.usc.edu/) on the genome region chr5: 149107388–149112388 corresponding to the putative has-miR378a-3p putative promoter.

Each experiment was repeated three times independently. All results were expressed as means ± standard deviation (SD), and a p-value < 0.05 was used for significance. One-way ANOVA analysis for independent samples was used to determine statistical significance in different assays.

### Cell cultures

Human ARMS RH30 and ERMS RD cell lines were maintained in high-glucose Dulbecco’s modified Eagle’s medium(DMEM-HG) supplemented with 10% foetal bovine serum (FBS), 1% v/v L-glutamine, 100 μg/ml streptomycin and 100 U/ml penicillin, and grown at 37°C in a humidified atmosphere of 5% CO_2_.

### 5-aza-2′-deoxycytidine treatment

RH30 and RD cells were seeded at 4 × 10^5^ cells/well in 6-well plates. After 24 h, 5-aza-dC (Sigma-Aldrich, St. Louis, MO) was added to a final concentration of 20 μM. Following different times of treatment, cells were collected for cell cycle analysis, apoptosis, MTT, migration, Q-PCR, western blot or immunofluorescence. Mock treatments were carried out treating cells in the same medium with DMSO (Ctr).

### Transient transfection

RH30 and RD cells were seeded at 8 × 10^5^ cells/well in 6-well plates; miRNA mimics (miR-378a-3p, Dharmacon Research) or negative control (miR-Ctr, Dharmacon Research) were transfected using Lipofectamine 2000 reagent (Life Technologies) at 50 nM final concentration, following the manufacturer’s protocol. Following different times of treatment, cells were collected for cell cycle analysis, apoptosis, MTT, migration, Q-PCR, western blot or immunofluorescence.

### Quantitative Real Time PCR (Q-PCR)

Reverse transcription (RT) for human miR-378a-3p, miR-378a-5p, miR-483-3p and miR-503-5p was carried out with TaqMan MicroRNA Assay kit (Life Technologies) using 20 ng of total RNA sample and the specific stem-loop primer according to manufacturer’s protocols. Quantitative Real Time PCR (Q-PCR) analysis was performed on a StepOne Real Time System (Life Technologies) machine using miRNA-specific TaqMan MGB primers/probe (Life Technologies). PCR reactions were run at 95°C for 10 min, followed by 40 cycles at 95°C for 15 s and 60°C for 30 s. Data were normalized to U6 small nuclear RNA (RNU6) levels. Each sample was run in triplicate. The amount of each miRNA was calculated by the comparative Ct method and expressed as fold change (2^-ΔΔCT^) compared to NSM using the DataAssist v3.01 software (Life Technologies).

### MTT assay

RH30 cells were treated with 5-aza-dC or transfected with miRNA mimics, and cell viability was determined using MTT [3-(4,5-dimethylthiazol-2-yl)-2,5-diphenyltetrazolium] assay. Cells (5×10^3^) were plated onto 96-well plates in sextuplicates and, after 24 h, treated with 5-aza-dC or transfected with synthetic miR-378-3p; untreated and blank cell-free controls were included. At designated times after treatment or transfection (0-24-48-72 h), 10 μl of MTT (5 mg/mL, Sigma-Aldrich) were added to each well and plates were incubated at 37°C for 4 h. Media were removed and 150 μl dimethyl sulfoxide (DMSO) were added into each well to dissolve the dark blue formazan crystals. Absorbance was measured at wavelength of 550 nm, with reference at 630 nm, using a microtitre plate reader (Select Science). The results were plotted as means ± SD of two separate experiments having six determinations per experiment for each experimental condition.

### Cell cycle analysis

RH30 and RD cells treated with 5-aza-dC were collected and washed twice with phosphate buffered saline (PBS). After fixation in 70% ice-cold ethanol overnight at +4°C, cell pellets were washed twice with ice-cold PBS and treated with RNase A for 15 min at 37°C. Propidium iodide (PI) was added to each sample and DNA content was determined by collecting 10,000 events using a BD FACS Calibur Flow Cytometer (BD Biosciences). Data were analysed using CellQuest Pro software (BD Biosciences). Experiments were performed three times.

### Apoptosis analysis

Cell apoptosis was analysed by flow cytometry with PE Annexin V Apoptosis Detection Kit I (BD Pharmingen). Briefly, RH30 and RD cells were seeded overnight in 6-well plate and treated with 5-aza-dC or transfected with miRNA mimics for 72 h. Cells were washed twice in cold PBS and resuspended in 1x Annexin V Binding Buffer at a concentration of 1×10^6^ cells/ml. Cells were stained with Annexin V and 7-Amino-Actinomycin D (7-AAD) for 15 min at room temperature (RT) in darkness according to the manufacturer’s instructions. Annexin V and 7-AAD fluorescence intensities of control or treated samples were analysed using a BD FACSCalibur Flow Cytometer (BD Biosciences). Data were analysed using Cell Quest Pro software (BD Biosciences). Experiments were performed three times.

### Migration assay

RH30 cells were cultured in complete medium with 5-aza-dC or miRNA mimics for 72 h before plating 5×10^4^ cells per well into BD FalconTM Cell Culture Inserts with 8 μm pore polycarbonate filters (Falcon). Chambers with cells contained medium without serum, whilst the lower well had DMEM supplemented with 10% FBS, used as chemoattractant. After 24 h, migrated cells at the base of the inserts were fixed in 100% methanol and stained with 2% crystal violet dye. Cells were photographed under a light microscope at 20× or 40× magnifications; 8 randomly selected fields were examined and counted manually. The average number of migrated cells was calculated. Experiments were performed in triplicate and repeated twice.

### Western blot analysis

RMS cells were seeded overnight in 6-well plates. Cells were treated with 5-aza-dC or transfected with miRNA mimics for 72–96 h and lysed with Hepes buffer (20 mM Hepes, 250 mM NaCl, 0.1% Triton X-100, 2 mM EDTA, 10 μg/mL leupeptin, 10 μg/mL aprotinin, 0.5 mM phenylmethylsulfonyl fluoride, 4 mM sodium orthovanadate, 1 mM DTT). Total protein extracts (30 μg) were separated on 8-12% sodium dodecyl sulfate (SDS)-polyacrylamide gel (PAGE) and transferred onto polyvinylidene fluoride (PVDF) membranes (Millipore Corporation, Bedford, MA). Filters were blocked with 5% non-fat dry milk in PBS-Tween for 30 min at RT and incubated with the primary antibody. The following antibodies were incubated over-night at +4°C: anti-IGFR1 (Cell Signaling), anti-phospho-Akt (Cell Signaling), anti-MyoR(Santa Cruz Biotechnology), anti-MyOD1 (Millipore), anti-Myf5 (Millipore), anti-desmin (Millipore) and anti-MyHC (Millipore). Appropriate horseradish peroxidase (HRP)-conjugated secondary antibodies (Santa Cruz Biotechnology) were used for 1 h at RT. Protein-antibody complexes were detected with ECL Super Signal (Pierce). Tubulin (Sigma-Aldrich) was used as a normalization control for equal loading. Experiments were performed at least three times.

### Immunofluorescence

RMS cells were seeded overnight in 24-well plates. Cells were treated with 5-aza-dC or transfected with miRNA mimics for 72 h and then seeded into 8-chamber culture slides (Falcon). After two additional days, cells were rinsed with PBS and fixed with 4% paraformaldehyde at RT for 30 min. After treatment with 0.1 M glycine and permeabilisation with 0.1% Triton X-100, cells were subjected to immunofluorescence staining with the anti-MyHC (Millipore) antibody for 1 h 30 min at RT. Cells were washed with cold PBS three times and incubated with Texas Red-anti-mouse secondary antibody (1:100, Jackson Laboratories) at RT for 30 min. The actin cytoskeleton was visualized with TRITC-phalloidin (1:50, Sigma) at RT for 1 h 30 min. Nuclei were counter-stained with 1 μg/ml Hoechst (Sigma). Labelled sections were examined and analysed by using a Zeiss ApoTom epifluorescent microscope (Carl Zeiss) and Axio-Vision software. Experiments were replicated twice.

### DNA Methylation analysis

Genomic DNA from RH30 cells treated or not with 5-aza-dC for 72 h was obtained by phenol:chloroform:isoamyl alcohol method. About 1 μg of DNA was modified using the Epitect DNA Bisulfite Kit (Qiagen), according to the manufacturer’s protocol. For sequencing, the bisulfite-treated DNA was amplified by PCR with two different primer sets for the human miR-378a promoter: BS1 forward 5′-GGGGAAAAGttAGGtTGGA-3′ and BS1 reverse 5′-aCTaACATTTTTaaTaaCTaCTTaTCCCAaC-3′; BS2 forward 5′-GGGTAAtTGGGGGTTttAG-3′ and BS2 reverse 5′-CAaCAACAaCACTCTaaaaACT-3′. PCR products, purified using a commercially available kit (Qiagen), were sequenced on an ABI sequencer with dye terminators (Applied Biosystems). Analysis of primary bisulfite sequencing was carried out with BISMA software (http://services.ibc.uni-stuttgart.de/BDPC/BISMA) by uploading the unconverted reference sequence and the sequencing results. For methylation-specific PCR (MSP), the bisulfite-modified genomic DNA was amplified using primers based on methylated (M) or unmethylated (UM) cytosines in CpG islands in the miR-378a promoter region: M forward 5′-AGtTAGCGGtttTGCGGtAGtC-3′ and M reverse 5′-aCCCGaaaaaAaaaAaCCAaCGAaCG-3′; UM forward 5′-tttGtttttGtAGtTAGtGGtttTGtGGtAGttG-3′ and UM reverse 5′-CaAaCCaaCCCaaaaaaAaaaAaCCAaCaAaCa-3′. PCR products were run on 2% agarose gel. All primers were designed using the MSPprimer algorithm. Experiments were performed three times.

## Results

### Small RNA library generation, sequencing, identification and quantification of annotated miRNAs

**Figure 1:**
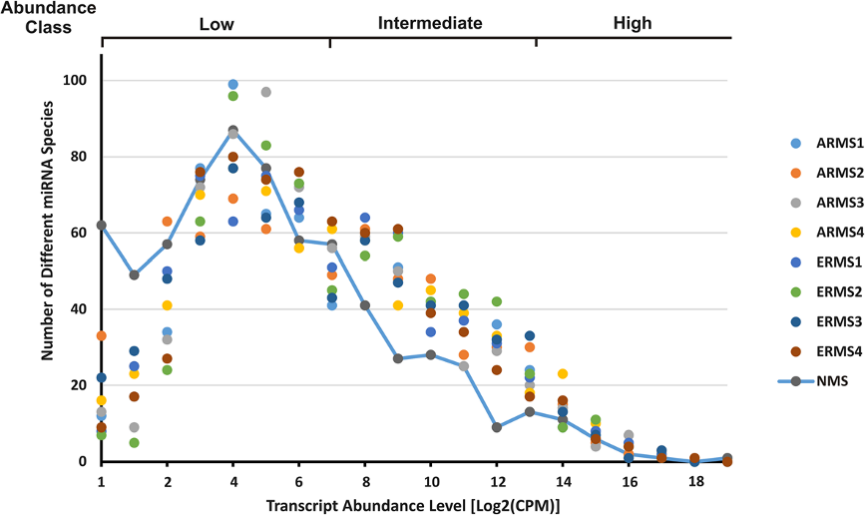
**miRNAs in alveolar and embryonal RMS (ARMS and ERMS) samples, together with normal skeletal muscle (NSM).** Graphic of the number of different miRNA species in function of transcript expression levels, expressed in Log_2_CPM, in ARMS, ERMS and NSM samples. Distribution of transcripts in abundance classes (Low < 1-10^2^ CPM; Intermediate 10^2^-10^4^ CPM; High > 10^4^ CPM) is schematized.

As accumulating evidence indicates that miRNAs play important roles in cancer development, including RMS, this study profiled the miRNA transcriptome by the direct sequencing of mature miRNA molecules in a panel of primary RMS tumours. RNA was prepared from four ARMSs and four ERMSs, and from a pool of NSM obtained from eight donors. As RMS has a mainly infiltrative growth pattern, the quantity of available tumour samples is very often a limiting factor, especially for controls and NGS experiments. Hence, we used a set of pre-pooled NSM samples from already available normal donors thus averaging out the variance at the expense of some loss in biological variability. However, relevant differential representation of single miRNA molecules stood out clearly in a comparison of individual samples versus a pooled control, minimizing the inter-individual ‘transcriptional noise’. Clinical characteristics of the tumour cases are reported in Table 1. Small RNA libraries were prepared and deep sequenced by using a SOLiD4 Sequencer platform. The corresponding nine cDNA libraries yielded a total of 250 million sequenced reads, and more than 85% of these reads (an average of 22 million per library) mapped against the human reference genome (GRCh37/hg19, repeat masked). The reads corresponding to annotated miRNAs were identified by selecting all reads mapping against the human precursor and mature sequences comprised in miRBase v19.0. After passing alignment quality filtering, from 2 to 7 million reads were identified as annotated miRNAs per library (on average 15% of hg19 mapped reads), representing 685 different mature miRNA molecules. The abundance value of each target-miRNA was normalized using TMM normalization, scaled to “counts per million” (CPM) in respect to each library size and the associated “pseudo-counts” were used for the differential expression analysis. Figure 1 and Table 2 show the distribution of the different miRNA species in abundance classes comparing ARMS and ERMS samples against NSM, allowing a survey of the whole miRNA population. Apparently, tumour miRNAs on average were more represented in the intermediate abundance class (10^2^-10^4^ CPM), whilst they were under-represented in the lowest (1-10^2^ CPM) and in the highest abundance (>10^4^ CPM) classes. These data support the hypothesis that in these tumours a rearrangement of miRNA expression levels occurred, resulting in a reduction of the levels of some highly expressed miRNAs and a simultaneous slight increase of the expression of many low-abundance miRNA species.

**Table 2:** **Distribution in abundance classes of different miRNA transcripts in tumours and normal muscle**

		NMS			RHABDOMYOSARCOMAS		
CLASSES	CPM range	miRNA species	Cumulative CPM	% CPM	miRNA species	Cumulative CPM	% CPM
**LOW ABUNDANCE**	1 - 10^2^	498	9,663	1.0	369	9,288	0.9
**INTERMEDIATE ABUNDANCE**	10^2^ - 10^4^	163	214,726	21.5	296	451,671	45.2
**HIGH ABUNDANCE**	>10^4^	16	775,611	77.5	20	539,041	53.9
**TOTAL**		677	1,000,000	100.0	685	1,000,000	100.0

The distribution of miR-387a-3p isomirs between NSM and RMSs was assessed. One YM500 database isoform with an extension at 3′ and a mismatch in the extension (ACTGGACTTGGAGTCAGAAGG**CG[C]T**) was represented in both NSM and ARMS/ERMS samples, but with a noticeable difference in frequency which will warrant further investigation.

### Differentially expressed miRNAs

Of a total of 685 expressed miRNAs, 97 (14.2%) displayed significant differential levels collectively in ARMS and ERMS tumours in comparison with NSM. Notably, out of these 97, 79 (81.4%) miRNAs were expressed at lower levels in RMSs using the TMM normalization (Table 3A), whilst 18 (18.6%) were expressed at higher levels (Table 3B). Of note, using a different edgeR normalization method (upper quartile) only produced 4 additional differentially expressed miRNAs, while maintaining the total number of detected small RNAs. Among the identified differentially expressed miRNAs, some have been previously associated with tumour development in RMS [36]. Interestingly, miRNAs belonging to the miR-378 family, recently suggested to be essential in normal skeletal muscle development [37], were markedly down-regulated in both ARMS and ERMS tumours (see Additional file 1: Table S1). MiR-133a, miR-378a-3p, miR-378a-5p, miR-483-3p and miR-503-5p were selected as candidates to validate miRNA expression levels in Q-PCR using the eight deep-sequencing-analysed RMSs, seven additional tumour samples (3 ARMSs and 4 ERMSs) along with four different RMS cell lines (RH4 and RH30 ARMS cell lines; and RD and RD18 ERMS cell lines). In agreement with the deep-sequencing findings, Q-PCR results confirmed the down-regulation of miR-133a, miR-378a-3p and miR-378a-5p, as well as the over-expression of miR-483-3p and miR-503-5p in the RMS tumour tissues (see Additional file 2: Figure S1A) and cells (see Additional file 2: Figure S1B). Comparison of deep sequencing ARMS and ERMS data showed a similar range of gene expression profile with the exception of a limited number of miRNAs that displayed significantly different levels (Table 3C).

**Table 3:** **miRNA expression in RMS tumours**

A						B		
**miRNA_ID**	**log**_**2**_ **FC**	**log**_**2**_ **CPM**	**miRNA_ID**	**log**_**2**_ **FC**	**log**_**2**_ **CPM**	**miRNA_ID**	**log**_**2**_ **FC**	**log**_**2**_ **CPM**
hsa-miR-885-5p	−6.93	7.34	hsa-miR-4256	−2.95	9.13	hsa-miR-4327	9.95	3.61
hsa-miR-144-5p	−5.62	8.27	hsa-miR-218-5p	−2.95	11.07	hsa-miR-508-3p	9.63	3.40
hsa-miR-451a	−5.52	15.36	hsa-miR-126-3p	−2.93	13.30	hsa-miR-383	7.99	6.17
hsa-miR-1	−5.47	16.81	hsa-miR-5787	−2.93	3.93	hsa-miR-503-5p	5.68	9.48
hsa-miR-378a-5p	−5.34	11.64	hsa-miR-22-5p	−2.88	9.77	hsa-miR-154-3p	5.19	10.09
hsa-miR-223-3p	−5.12	12.89	hsa-miR-30e-3p	−2.81	9.06	hsa-miR-493-5p	4.89	11.33
hsa-miR-124-3p	−5.06	5.79	hsa-miR-139-5p	−2.79	9.02	hsa-miR-450a-5p	4.88	9.11
hsa-miR-378a-3p	−4.92	13.79	hsa-miR-30b-3p	−2.77	3.39	hsa-miR-500b	4.68	9.25
hsa-miR-378f	−4.80	7.02	hsa-miR-203a	−2.72	5.26	hsa-miR-335-3p	4.60	9.51
hsa-miR-378 h	−4.53	4.42	hsa-miR-185-5p	−2.71	8.80	hsa-miR-376c-5p	4.59	4.14
hsa-miR-378c	−4.47	12.37	hsa-miR-652-3p	−2.68	7.09	hsa-miR-483-3p	4.55	13.10
hsa-miR-599	−4.39	4.93	hsa-miR-4484	−2.64	3.45	hsa-miR-376a-2-5p	4.55	5.55
hsa-miR-144-3p	−4.36	7.41	hsa-miR-208b	−2.63	5.74	hsa-miR-503-3p	4.55	4.22
hsa-miR-378i	−4.29	6.47	hsa-miR-24-1-5p	−2.60	5.69	hsa-miR-3182	4.43	7.92
hsa-miR-378d	−4.29	8.81	hsa-miR-30a-3p	−2.60	8.81	hsa-miR-542-3p	4.36	10.77
hsa-miR-486-5p	−4.14	11.78	hsa-miR-29a-3p	−2.59	13.25	hsa-miR-149-5p	4.33	11.39
hsa-miR-133a	−4.05	14.25	hsa-miR-16-2-3p	−2.55	8.81	hsa-miR-501-5p	3.81	11.03
hsa-miR-95	−3.96	9.35	hsa-miR-628-3p	−2.54	2.47	hsa-miR-483-5p	3.48	9.68
hsa-miR-378b	−3.86	5.55	hsa-miR-29b-2-5p	−2.54	5.75			
hsa-miR-29c-5p	−3.82	7.35	hsa-miR-133b	−2.53	11.85	**C**		
hsa-miR-378e	−3.82	9.31	hsa-miR-4300	−2.51	4.76	**miRNA_ID**	**log**_**2**_ **FC**	**log**_**2**_ **CPM**
hsa-miR-142-5p	−3.68	8.68	hsa-miR-491-5p	−2.32	2.56	hsa-miR-335-5p	−3.25	12.19
hsa-miR-4461	−3.66	2.84	hsa-miR-873-3p	−2.31	3.06	hsa-miR-489	−3.30	7.64
hsa-miR-378 g	−3.64	4.58	hsa-miR-3687	−2.31	2.75	hsa-miR-375	−4.11	8.33
hsa-miR-944	−3.56	2.73	hsa-miR-5095	−2.27	4.86	hsa-miR-135a-5p	−5.38	10.49
hsa-miR-486-3p	−3.54	6.75	hsa-miR-29b-1-5p	−2.26	3.58	hsa-miR-9-3p	−6.07	6.89
hsa-miR-29c-3p	−3.54	12.27	hsa-miR-664a-5p	−2.25	3.52	hsa-miR-9-5p	−7.53	8.95
hsa-miR-4306	−3.53	3.61	hsa-miR-664a-3p	−2.24	7.74	hsa-miR-499a-3p	6.21	7.83
hsa-miR-4516	−3.51	3.57	hsa-miR-4315	−2.22	4.39	hsa-miR-499a-5p	6.07	9.97
hsa-miR-193b-5p	−3.51	6.08	hsa-miR-3714	−2.21	2.64	hsa-miR-432-5p	3.58	9.47
hsa-miR-139-3p	−3.49	2.94	hsa-miR-4787-3p	−2.18	2.50	hsa-miR-296-5p	3.37	8.53
hsa-miR-616-5p	−3.41	4.29	hsa-miR-7-5p	−2.14	6.83	hsa-miR-95	3.30	7.89
hsa-miR-22-3p	−3.39	9.05	hsa-miR-7-1-3p	−2.13	7.81	hsa-miR-31-5p	3.22	8.09
hsa-miR-150-5p	−3.33	10.54	hsa-miR-7-2-3p	−2.06	3.97			
hsa-miR-548v	−3.27	3.04	hsa-miR-23c	−1.98	9.82			
hsa-miR-3117-3p	−3.05	1.97	hsa-miR-584-5p	−1.96	2.87			
hsa-miR-628-5p	−3.03	5.41	hsa-miR-137	−1.95	5.54			
hsa-miR-1281	−3.02	3.73	hsa-miR-3607-5p	−1.64	10.85			
hsa-miR-548z	−3.01	3.45	hsa-miR-935	−1.59	5.49			
hsa-miR-126-5p	−3.00	12.54						

(A) miRNA species expressed at lower levels in RMSs *vs.* NSM (ARMS and ERMS are collectively considered); (B) miRNA species expressed at higher levels in RMSs *vs.* NSM (ARMS and ERMS are collectively considered); (C) differentially expressed miRNAs in ERMS *vs.* ARMS tumours.

### miR-378a-3p negatively regulates IGF1R levels

**Figure 2:**
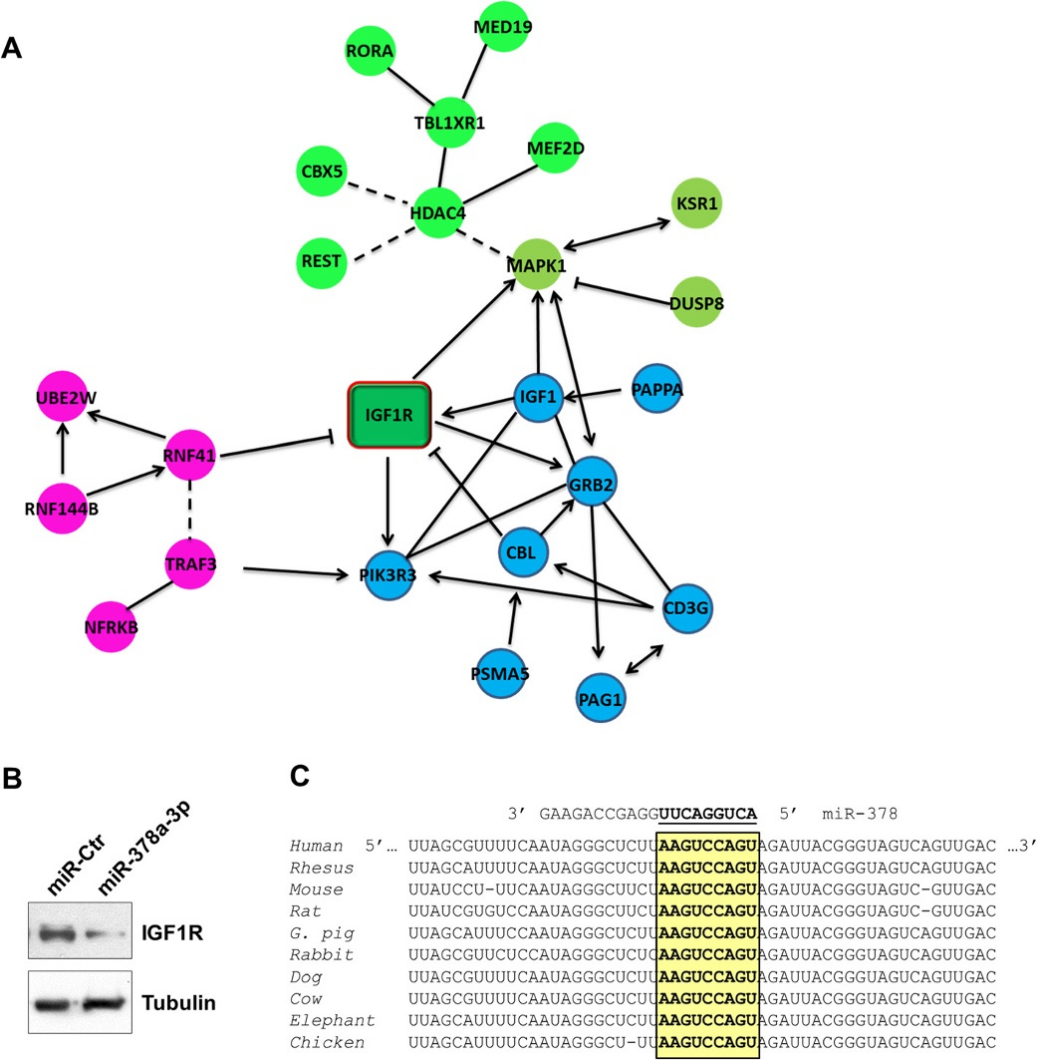
**miR-378a-3p negatively regulates IGF1R. (A)** Schematic network representation of miR-378a-3p target genes functional annotations and interactions obtained from Cytoscape Reactome FI interrogation. IGF1R represents the principal mRNA target of miR-378a-3p. Putative mRNA targets of miR-378a-3p are predicted to be involved in several signal pathways, specifically apoptosis, cell cycle and chromatin remodelling. Arrow (edge) orientation indicates positive, negative or undetermined functional interaction between different genes (network nodes). Target gene colours indicate different functional modules of the interaction network. **(B)** Immunoblot analysis. Validation of miR-378a-3p direct participation in the post-transcriptional regulation of IGF1R in RH30 cells (miR-control *vs.* miR-378a-3p positive cells). Tubulin was used as loading control. **(C)** Sequence alignment of miR-378a-3p seed sequence (highlighted) with IGF1R 3′-UTR binding sites (bold letters in the yellow box; position 5589–5596 of human IGF1R: NM_000875) in different species. Data from TargetScan Human 6.2 interrogation.

As reported in Table 4, miR-378 family members are transcribed from different loci but they exhibit seed sequence homology for mRNA target recognition. We focused on miR-378a-3p, a key regulatory molecule of miR-378 family, investigating the biological relevance of this miRNA in RMS tumours by performing target gene prediction and functional pathway analysis. TargetScan, DIANA-microT, miRanda and miRWalk algorithms were interrogated, which allowed the identification of a list of putative/validated mRNA targets for miR-378a-3p (see Additional file 3: Table S2). DAVID and Cytoscape Reactome FI, used to functionally cluster the miRNA targeted transcripts and their involvement in various signal pathways, showed a significant enrichment in biological mechanisms and pathways linked with neoplastic diseases and, more specifically, with apoptosis, cell cycle signalling and DNA remodelling/interaction (see Additional file 3: Table S2). Interestingly, the *in silico* analysis suggested that miR-378a-3p directly participates in the post-transcriptional regulation of IGF1R signalling (Figure 2A), which is involved in the development, growth, proliferation, cell survival and metastasis of RMS [38]. To validate the control of the IGF1R expression, transient transfection ofmiR-378a-3p in RH30 cells, an *in vitro* model of human ARMS, was carried out. An RNA duplex from *C. elegans* was used as a negative miRNA-Control (miR-Ctr). Using by stem-loop real-time PCR, specific expression increase of miR-378a-3p after transfection with the respective mimic was demonstrated (see Additional file 4: Figure S2). A down-regulation of the endogenous IGF1R protein levels was observed in miR-378a-3p-transfected RH30 cells (Figure 2B) consistent with the conserved binding sites for this miRNA in the 3′-untranslated region of IGF1R transcript (Figure 2C), identified by computational tools for miRNA target prediction. IGF1R reduction was also confirmed in RD cells transiently transfected with miR-378a-3p (data not shown). These data are also in agreement with those reported by other research groups [39–41] in which the direct regulation of IGF1R mRNA by miR-378a-3p was supported by *in vitro* luciferase assays. Thus, expression of miR-378a-3p suppresses IGFR1 pathway activity.

**Table 4:** **Human miR-378 family members**

miRNA_ID	Accession	Sequence	Locus (strand)	Start	End
hsa-miR-378a-3p	MIMAT0000732	ACUGGACUUGGAGUCAGAAGG	Chr5 (+)	149112388	149112453
hsa-miR-378b	MIMAT0014999	ACUGGACUUGGAGGCAGAA	Chr3 (+)	10371913	10371969
hsa-miR-378c	MIMAT0016847	ACUGGACUUGGAGUCAGAAGAGUGG	Chr10 (−)	132760851	132760931
hsa-mir-378d-1	MIMAT0018926	ACUGGACUUGGAGUCAGAAA	Chr4 (−)	5925002	5925055
hsa-mir-378d-2	MIMAT0018926	ACUGGACUUGGAGUCAGAAA	Chr8 (−)	94928250	94928347
hsa-miR-378e	MIMAT0018927	ACUGGACUUGGAGUCAGGA	Chr5 (+)	169455492	169455570
hsa-miR-378f	MIMAT0018932	ACUGGACUUGGAGCCAGAAG	Chr1 (+)	24255560	24255637
hsa-miR-378 g	MIMAT0018937	ACUGGGCUUGGAGUCAGAAG	Chr1 (−)	95211416	95211456
hsa-miR-378 h	MIMAT0018984	ACUGGACUUGGUGUCAGAUGG	Chr5 (+)	154209018	154209100
hsa-miR-378i	MIMAT0019074	ACUGGACUAGGAGUCAGAAGG	Chr22 (−)	42319226	42319301
hsa-miR-378j	MIMAT0024612	ACUGGAUUUGGAGCCAGAA	Chr17 (−)	35974976	35975084
hsa-miR-378a-5p	MIMAT0000731	CUCCUGACUCCAGGUCCUGUGU	Chr5 (+)	149112388	149112453

MiRBase (Release 21, June 2014) identifiers, accession numbers, sequence and genome location of human miR-378 family members. The presence of a shared common seed is evident from the aligned sequences.

### miR-378-3p induces apoptosis and impairs cell migration

**Figure 3:**
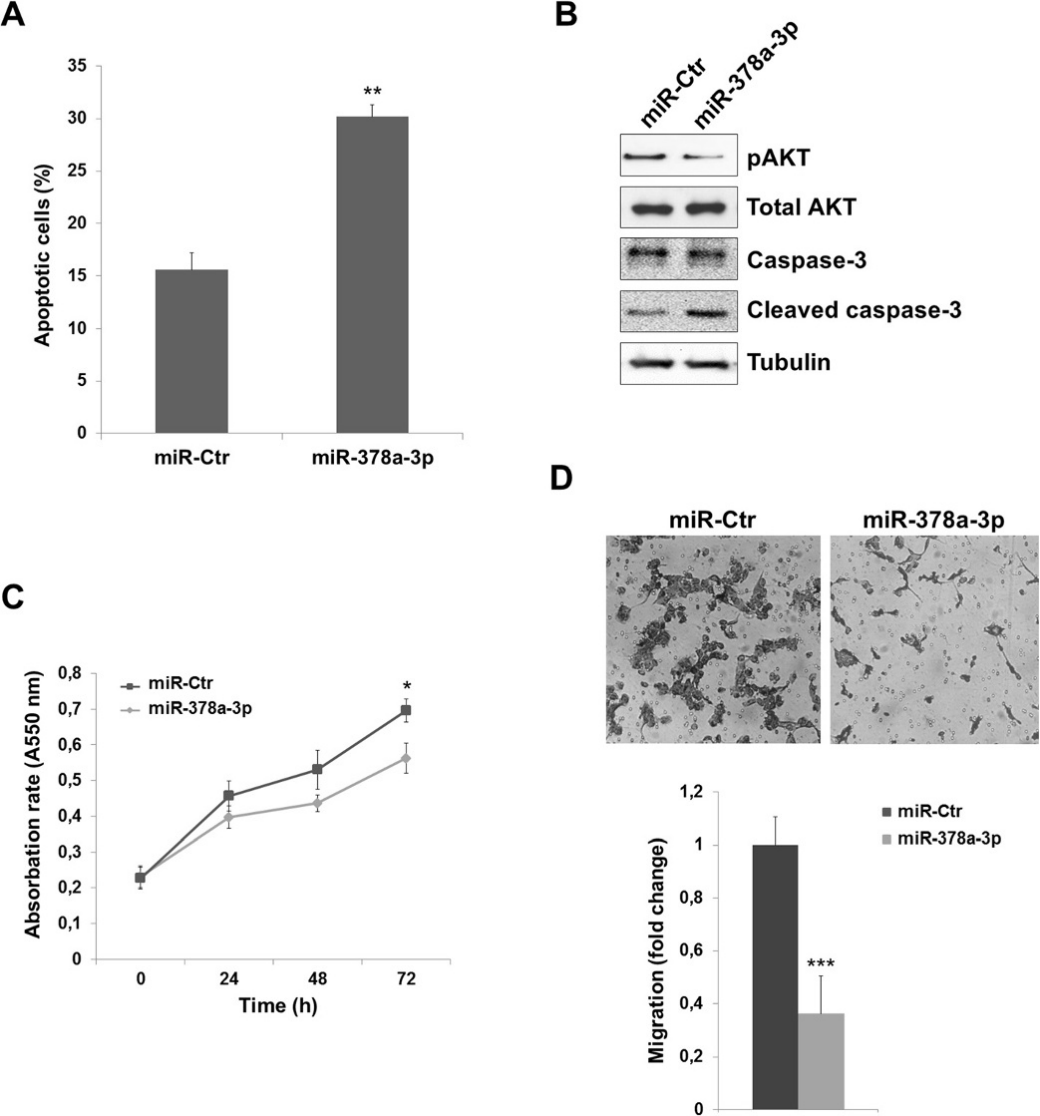
**miR-378a-3p involvement in apoptosis, cell proliferation and migration of RMS cells. (A)** FACS analysis. Increase of apoptotic cells (Annexin V-positive) in miR-378a-3p compared to miR-Control positive RH30, 72 h after transfection. Results in the bar chart are the means ± standard deviation (SD) of three separate experiments. **(B)** Immunoblot analysis, 72 h after transfection. Reduction of phosho-AKT (pAKT) levels and up-regulation of cleaved caspase-3 in RH30 positive cells in comparison to miR-Ctr transfected cells. Total AKT andcaspase-3 levels were similar in both samples. Tubulin was used as loading control. **(C)** MTT assay. Alteration of the cell viability/proliferation rate in miR-378a-3p transfected RH30 compared with mock control cells. Results are means ± SD of sextuplicate points of two separate experiments. **(D)** Migration assay. miR-378a-3p transfected RH30 cells compared to negative control cells showed decreased ability to migrate through Boyd chamber membranes. Representative fields of migrated cells through the membrane, stained with crystal violet dye (20× magnification). Data was obtained from the average migration cell number per field (n =8) ± SD of triplicate experiments performed twice. Asterisks denote data in which miR-378a-3p was significantly different from control (*, p < 0.05; **, p < 0.01; ***, p < 0.001).

In order to examine the potential anti-oncogenic role of miR-378a-3p in RMS, a series of *in vitro* gain-of-function experiments were designed. A significant increase of apoptotic cells was observed in alveolar RH30 at 72 h following transfection of miR-378a-3p mimics (30.8% ± 1.0) in comparison with miR-Ctr (16.3% ± 2.4) (Figure 3A). Similarly, the number of apoptotic cells significantly increased in miR-378a-3p transfected embryonal RD cells *versus* miR-Ctr positive cells (24.1% ± 2.9 *vs.* 11.2% ± 1.9, p < 0.01). Consistent with the FACS results, a decrease of AKT phosphorylation levels and a concomitant increase of cleaved-caspase-3, an important regulator of apoptosis, were detected in miR-378a-3p positive cells compared to scrambled control-treated RH30 cells (Figure 3B). Only a moderate alteration (about 20%) in cellular viability/proliferation rate was evident in miR-378a-3p-transfected RH30 cultures (Figure 3C). Ectopic expression of miR-378a-3p significantly suppressed by about 60% the ability of RH30 cells to migrate through Boyden chamber membranes towards serum-containing medium when compared with a mimic negative control (Figure 3D). These results indicate that the restoration of miR-378a-3p levels enhances programmed cell death and inhibits cell viability and migration potentials of RMS cells.

### miR-378a-3p correlates with myogenic differentiation

**Figure 4:**
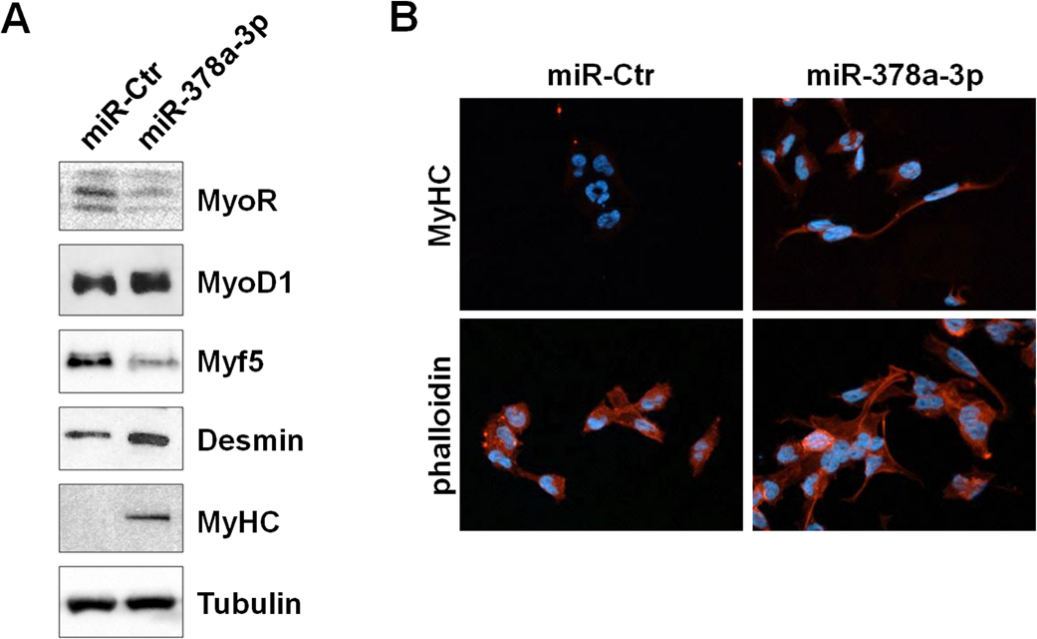
**miR-378a-3p activates myogenic differentiation. (A)** Immunoblot experiments. RH30 cells transfected with miR-378a-3p mimics or miR-Ctr were analysed for the expression of myogenic differentiation markers. Increased levels of MyoD1 (1.6-foldchange by densitometry), desmin and MyHC proteins and down-regulation of MyoR and Myf5 factors were evident. Tubulin served as protein loading control. **(B)** Immunofluorescence analysis. MiR-378a-3p ectopic expression evoked noticeable changes in RH30 cell morphology, a more organized actin and myosin arrangement, as visualised by TRITC-phalloidin staining, and a significant positivity for anti-MyHC antibody (100× magnification).

Expression analysis was carried out using a panel of muscle markers in RH30 cells transfected with miR-378a-3p in order to assess whether miRNA up-regulation was able to promote skeletal muscle differentiation. The modifications found in specific myogenic marker levels were consistent with the induction of myogenic differentiation both in immunoblotting and immunofluorescence experiments, performed 96–120 h after miR-378a-3p induction (Figure 4). In particular, RH30 cells transfected with miR-378a-3p mimics showed a slight up-regulation of MyoD1 and MyHC proteins, which are respectively detected in committed proliferating myoblasts and in post-mitotic muscle cells, with a concomitant down-regulation of MyoR, a repressor of myogenesis, and Myf5, an helix-loop-helix transcription factor correlated with myoblast proliferation stage (Figure 4A). Myoid differentiation was also confirmed by the up-regulation of the intermediate filament desmin, the contractile protein actin expressed in skeletal muscle cells (Figure 4A). Furthermore, an increased number of miR-378a-3p transfected cells exhibited changes in morphology/cytoskeleton organization and moderate levels of MyHC staining (Figure 4B). In particular, more organized actin filaments were observed in miR-378a-3p positive cells, whilst actin appeared more diffusely distributed through-out the cytoplasm in mocked RH30 control cells, as documented by TRITC-phalloidin staining (Figure 4B). MyHC immunofluorescence staining had stronger intensity also in RD cells transfected with miR-378a-3p than miR-Ctr (data not shown). These experiments suggest that miR-378a-3p molecules are involved in the reactivation of terminal myogenic differentiation in RMS by coordinating specific regulatory factors and repressors.

### 5-aza-2′-deoxycytidine (5-aza-dC) treatment up-regulates miR-378a-3p levels and induces apoptosis, G2-cell arrest and decreased migration in RMS cells

**Figure 5:**
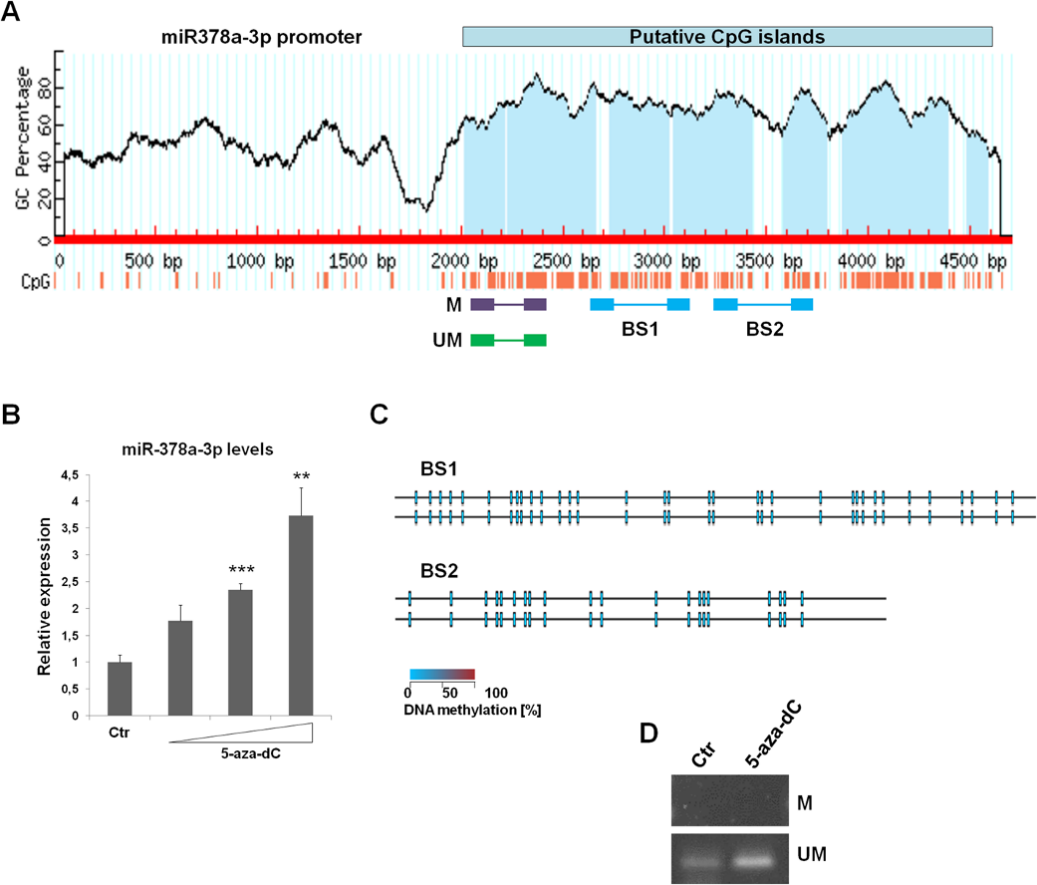
**miR-378a-3p response to 5-aza-dC treatment. (A)** Schematic representation of the miR-378a-3p promoter region generated by EMBOSS software. Bioinformatic analysis showed the presence of a high percentage of GC nucleotides and an enriched zone of CpG islands. **(B)** Effect of increasing doses of 5-aza-dC (2-10-20 μM) on mature miR-378-3p levels compared with control RH30 cells 24 h after treatment. Results are means ± SD of three separate experiments (**, p < 0.01; ***p < 0.001). **(C)** BISMA diagrams showing the methylation level of CpG dinucleotides in the miR-378a promoter in 5-aza-dC and mocked control cells, as revealed by bisulfite sequencing of BS1 and BS2 regions. Blue rectangles indicate non-methylated CpGs. **(D)** Methylation analysis of miR-378a promoter region by PCR using primer set for methylated (M) or unmethylated (UM) sequences. The presence of a visible UM product indicates that CpG dinucleotides are not methylated in both Ctr and 5-aza-dC-treated cells.

**Figure 6:**
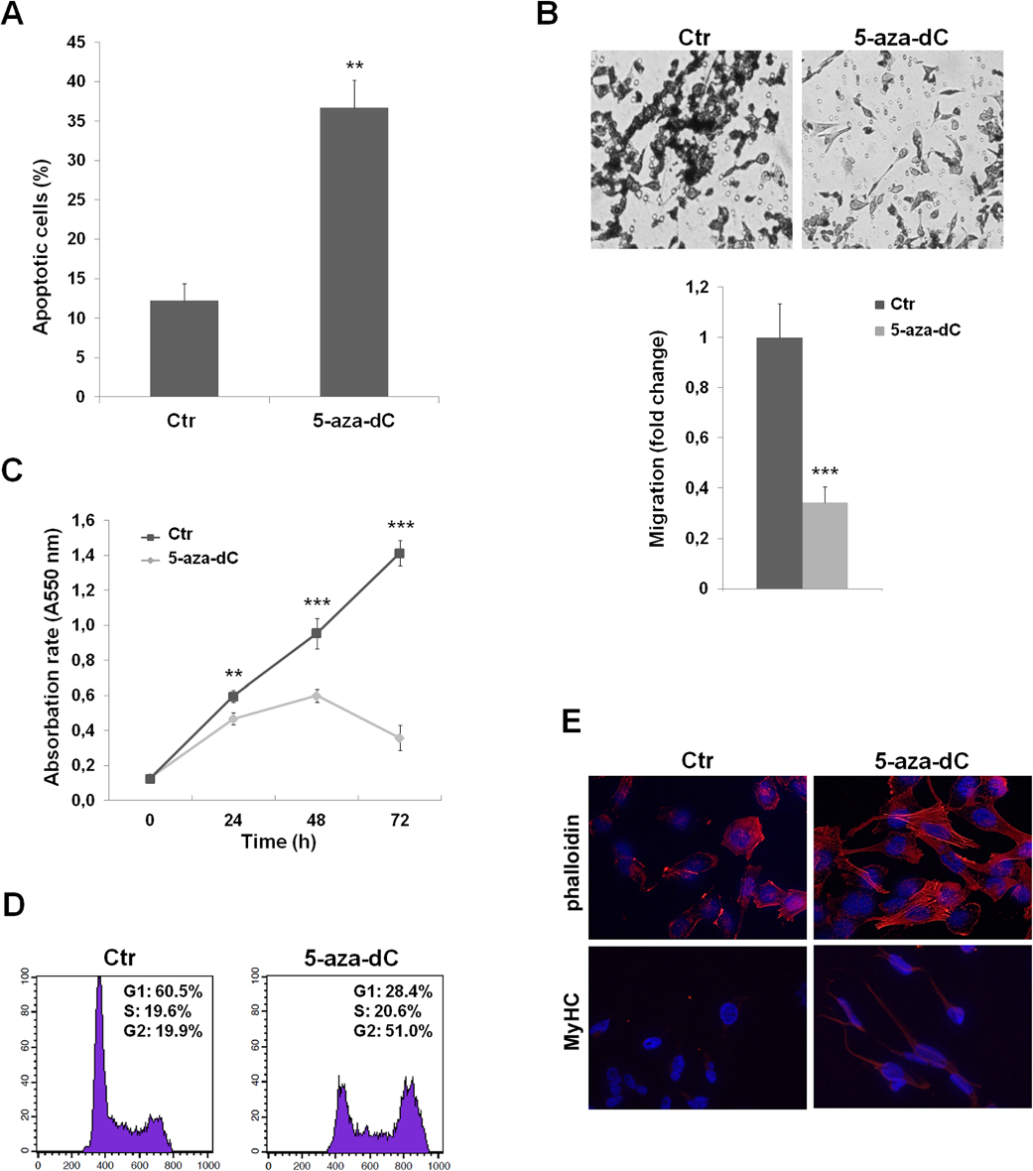
**5-aza-dC involvement in apoptosis, cell proliferation, migration and differentiation of RMS cells. (A)** FACS analysis. Increased number of apoptotic RH30 cells after treatment with 20 μM 5-aza-dC along with paired negative controls (DMSO treatment). Results are the means ± SD of three separate experiments. **(B)** Migration assay. 5-aza-dC treated RH30 cells, compared with control, showed significantly impaired ability to cross through Boyd chamber membranes. Representative fields of migrated cells through the membrane, stained with crystal violet dye (20× magnification). **(C)** MTT assay. Time-dependent inhibition of cell proliferation after 5-aza-dC treatment resulted in 80% decrease at 72 h. Data are shown as means ± SD of sextuplicate points of two separate experiments. **(D)** FACS analysis. Cell cycle distribution of 20 μM 5-aza-dC RH30 treated cells showed a marked arrest in G2-phase after 72 h. Results are means ± SD of three separate experiments. **(E)** Immunofluorescence analysis. Five-aza-dC exposure led to marked changes of RH30 cell morphology, to more organised actin bundles (TRITC-phalloidin staining) and to MyHC positivity (100× magnification). Asterisks denote data in which miR-378a-3p was significantly different from control (**, p < 0.01; ***, p < 0.001).

Since epigenetic modifications have been involved in miRNA deregulation, treatment of RH30 and RD cells with 5-aza-dC, a demethylating drug, was carried out to see if it could have a direct effect on miR-378a-3p levels given the presence of CpG islands in its promoter region (Figure 5A). Incremental dosing of 5-aza-dC (2-10-20 μM) resulted in up-regulation of the abundance of mature miR-378a-3p molecules compared with the control after 24 h (Figure 5B). A similar miR-378a-3p increment (about 3.5 fold-change) was observed in RD treated-cells (data not shown). In this investigation, we analysed the miR-378a-3p methylation pattern in RH30 cells, either untreated or treated with 5-aza-dC for 72 h. DNA was subjected to bisulfite sequencing (BS) and methylation-specific PCR (MS-PCR). Surprisingly, we had no evidence of methylated CpG islands either in Ctr (upper line) than in 5-aza-dC-treated cells (lower line) by sequencing BS1 and BS2 miR-378a-3p promoter regions (Figure 5C). Unmethylated pattern was also suggested by PCR positivity using primer sets specific for the miR-378a-3p unmethylated CpG (UM) but not with methylated CpG (M) specific primers (Figure 5D). The addition of 20 μM 5-aza-dC was able to efficiently trigger apoptosis in alveolar RH30 cells (36.7% ± 3.6 *vs.* 12.4% ± 2.2) (Figure 6A), whilst the amount of apoptotic cells was less in 5-aza-dC embryonal RD cells *versus* mocked control cells (26.1% ± 3.8 *vs.* 12.1% ± 2.0, p < 0.05). The migration capacity of the 5-aza-dC-treated RH30 cells was significantly diminished compared with a paired negative control (Figure 6B). Furthermore, a significant growth/cell cycle arrest of RH30 cells was evident after drug exposure. The addition of the demethylating drug resulted in the appearance of a small number of living cells with a different morphology compared to untreated control cells. A time-dependent inhibition of cell proliferation resulted in a peak of 80% at 72 h post-treatment in RH30 cells (Figure 6C). Similarly, 5-aza-dC reduced migration and proliferation rates of RD cells of about 50% (data not shown). To determine whether growth inhibition was associated with specific cell cycle changes, propidium iodide was added to RMS cells after 72 h of 5-aza-dC exposure. Cell cycle distribution analysis in RH30 cells showed a significant accumulation of cells in the G2 phase in 5-aza-dC treated cells compared with controls (51.% ±2.1 *vs.* 19.9% ±3.1, p < 0.001), while the percentage of cells in the G1 phase decreased by 50% (28.4% ±5.1 *vs*. 60.5% ±3.2, p < 0.01) (Figure 6D). Analogous trend was observed in RD cells treated with 5-aza-dC compared with mocked control (G2 phase: 50.2% ±1.1 *vs.* 35.9% ±1.7, p < 0.01; G1 phase: 30.1% ±0.9 *vs*. 47.5% ±1.3, p < 0.01).

These results indicate that demethylation inhibits viability and proliferation of RMS cells due to G2 cell cycle arrest and programmed cell death, and that it might also affect RMS progression by reducing cell migration.

### 5-aza-dC treatment induces myogenic differentiation

Finally, to assess whether 5-aza-dC treatment can also promote myogenic differentiation in RMS, RH30 cells were exposed to the demethylating drug followed by assessment of myogenic markers by immunofluorescence experiments. RH30 cells treated with 5-aza-dC exhibited marked changes in the cytoskeleton structure, as evident by phalloidin staining, and a differentiated phenotype, supported by the MyHC positivity (Figure 6E).

Taken together, these data are partially consistent with those following miR-378a-3p transfection, although the effects in RMS cells are more pronounced, this being due to the fact that 5-aza-dC action is not limited to the regulation of an individual miRNA but that it facilitates the re-expression of several epigenetically silenced genes.

## Discussion

RMS is a malignant tumour occurring most commonly in the paediatric population, with a survival of around 20% when metastatic disease is present at diagnosis [42]. MiRNAs seem to play a crucial role in RMS development and progression, and might represent novel tools for improving the outcome of this malignancy [12, 43, 44]. In this study, the expression levels of miRNAs were measured in a panel of alveolar and embryonal tumours by using deep sequencing technology in order to identify key drivers of RMS specific processes. The global miRNA abundance was reduced in RMS, this contributing to the neoplastic transformation by allowing an increased expression of proteins with oncogenic potential, as previously observed [17]. Notably, many relevant myo-miRNAs, such as miR-1, miR-133a and miR-29, were markedly down-regulated in tumour samples in comparison to NSM, as already shown in previous studies carried out by microarray or Q-PCR technologies [21, 37]. Interestingly, mir-206 was present with a detectable negative fold change in all comparisons but without being associated with a significant statistical index in the final analysis, as also reported in recent published microarray experiments carried out in the same biological model [45]. MiRNAs expression was relatively uniform in ARMS and ERMS tumours, and only a restricted number of small regulatory molecules seemed to discriminate the two RMS subtypes. Further analyses need to be undertaken to understand the possible differential role of specific miRNAs and related pathways in ARMS and ERMS oncogenic transformation, and to determine the potential role of miRNAs as diagnostic biomarkers in the two RMS subtypes.

To our knowledge, this is the first study that shows a marked down-regulation of miR-378 family members in RMS. This finding is probably due to the improved specificity and sensitivity of deep sequencing compared to microarray and Q-PCR analyses, deep sequencing also allowing identification of novel MiRNA isoforms. MiR-378 molecules were strongly under-expressed both in ARMS and ERMS tumours, this showing that the regulation of these miRNAs does not differentiate among RMS subtypes. Interesting preliminary results showed a significant difference in the abundance of one specific miR-378a-3p 3′ isoform between RMS and NSM. miR-378 molecules are encoded by different genes but they share identical seed sequences for mRNA target recognition. The most important regulatory small RNA of this family is miR-378a-3p, whose expression is highly variable in different cancers, reflecting the complexity and specificity of the miRNA response [46–48]. In addition, some studies suggest that miR-378a-5p, the opposite strand of miR-378a-3p, also has a functional relevance [49, 50]. Our data confirmed that miR-378a-3p was significantly reduced not only in RMS tumours but also in human RMS cell lines, this finding suggesting a role of miR-378a-3p as a tumour suppressor. Among the main pathways identified as potential targets for miR-378a-3p in our bioinformatics analysis, we chose to focus on IGF1R/AKT signalling, which has a well-established role in cellular growth and survival as well as in myogenic differentiation [40, 51, 52]. Ectopic expression of miR-378a-3p in RH30, an ARMS-derived cell line, markedly reduced IGF1R protein levels and promoted apoptosis via phospho-AKT inhibition and concomitant caspase-3 activation. Furthermore, miR-378a-3p-transfected cells moderately reduced cell proliferation and markedly cell migration. Accordingly, miR-378a-3p has been shown to be involved in carcinogenesis and an anti-oncogenic function has been suggested both in nasopharyngeal [53] and gastric cancer [47] through the negative regulation of VEGF expression, a protein promoting cell migration and inhibiting apoptosis. In a study by Martins et al. [54], RMS development seemed to involve increased IGF1R expression, which in turn enhanced AKT and Bcl-XL-mediated cell survival. The moderate effects on apoptosis and cell growth we observed in RH30 cells could be explained by the presence of the t(2;13)(q35;q14) translocation, since PAX3/FOXO1 chimeric protein is directly involved in the regulation of the IGF1R expression.

In accordance with the observation that in RMS proliferation and migration are inversely related to myogenic differentiation [3, 55], we found that miR-378a-3p mimic transfection was also able to promote myogenesis, as shown by the modulated levels of specific differentiation markers and by the changes in the cell morphology, from star-round to a more elongated appearance. In particular, normalization of miR-378a-3p expression reduced the levels of MyoR, a MyoD1 inhibitor, thus contributing to reactivate myogenesis; this was reflected by the MyHC levels, which is specifically related to committed muscle cells. Furthermore, miR-378a-3p transfected RH30 cells showed a more concentrated assembly of actin bundles, a characteristic of the intermediate muscle differentiation stage [56]. In accordance with these results, recent works have reported miR-378a-3p as a new candidate in skeletal muscle development [37, 57]. In C2C12 murine proliferating myoblasts, up-modulation of miR-378 has been proposed as allowing the efficient myotube formation by repressing antagonists of differentiation, like MyoR [57]. Likewise, the expression pattern of miR-378 in porcine longissimus muscles is closely related to myogenesis regulation, mainly with fibre formation [37]. Re-activation of skeletal muscle development in RMS seems to be linked to the IGF1R inhibition, as demonstrated by miR-1 and miR-133a [51, 58]. Indeed, the marked down-regulation of miR-378a-3p, miR-1 and miR-133a, and the concomitant up-regulation of IGF1R observed in RMS tumours could explain why cancer cells are prohibited from undergoing terminal differentiation despite their commitment to a myogenic pathway.

This study also underlines the relationship between aberrant epigenetic regulation of tumour suppressors, including miRNAs, and cancer development. The presence of CpG sites in the promoter regions of the miR-378 family members has recently been suggested and partially experimentally demonstrated in other neoplasms [47, 59–61]. In our *in vitro* RMS model, treatment with 5-aza-2′-deoxycytidine, a demethylating agent, was able to restore the expression of mature miR-378a-3p in a dose-dependent manner but without direct effect on DNA methylation status. Therefore, the hyper-methylation of CpG islands at the miR-378a-3p promoter region does not represent a causative mechanism for the marked down-regulation of this miRNA in RMS. Indeed, many CpG islands located at genes that have a tissue-restricted expression pattern, can remain methylation-free even when their associated genes are silent. Inactive unmethylated CpG island promoters show elevated levels of dimethylation at histone H3 lysine 4 (H3K4), suggesting that this chromatin mark may protect DNA from methylation [62]. In line with this model, recruitment of H3K4 methylases by unmethylated CpGs has recently been suggested [63]. Notwithstanding, our data imply that epigenetic modifications may play an indirect role on miR-378a expression by acting on specific transcription factors able to bind the miR-378a promoter region, which also warrant further study. Consistent with the up-regulation of miR-378a-3p levels, 5-aza-dC exposure affected apoptosis, proliferation and migration of RMS cells, with more dramatic effects. In particular, the resulting DNA hypomethylation arrested the cell cycle at G2-phase with a concomitant induction of myogenic differentiation, as demonstrated by the more elongated cell morphology and enhanced MyHC expression. Most likely, the fact that azacytidine has a much broader effect on the RH30 cellular phenotype reflects the fact that the transcriptional activation is not limited to miR-378a-3p but involves several epigenetically silenced genes. Aberrant DNA methylation and histone deacetylation was reported to have a key role in silencing plakoglobin gene expression in ARMS cells, whose over-expression was shown to suppress tumorigenicity in different cancer cell lines [64]. Moreover, recent findings suggest that suppression of BMP2 by epigenetic silencing may play a critical role in the genesis of RMS [65]. Based on these findings, 5-aza-dC might be a promising therapeutic agent in patients with RMS. To date, azacytidine is FDA-approved for the treatment of patients with acute myeloid leukemia and myelodysplastic syndrome [66, 67]. Data in sarcomas are still limited [68], but ongoing clinical trials in the field of epigenetic treatment suggest that this approach may be a potential option for patients with sarcomas, including RMS [69].

## Conclusions

This study suggests that miR-378a-3p is involved in RMS pathogenesis, exerting tumour suppressor-like activity. Since miR-378a-3p is down-regulated in other malignancies, the identification of the regulated tumorigenic pathways might have important implications in oncology. A better understanding of the biological mechanisms underlying RMS development represents a fundamental step in order to identify novel diagnostic and prognostic markers, as well as to develop targeted therapies in children and adolescents with RMS. Indeed, a combination of molecules inhibiting IGF1R signalling and promoting demethylation might be a possible therapeutic strategy worth to be further investigated in this tumour.

## Electronic supplementary material

Additional file 1: Table S1: Expression of miR-378 family members in RMS tumours. (A) miR-378 molecules differentially expressed in ARMs *vs.* NMS, with NMS baseline. (B): miR-378 molecules differentially expressed in ERMS *vs.* NMS, with NMS baseline. The members of the miR-378 family have FC values always negative and significant in both comparisons, indicating a strong under-expression in the investigated ARMS and ERMS samples. (DOC 43 KB)

Additional file 2: Figure S1: Validation of miRNA array data by quantitative real-time polymerase chain reaction (Q-PCR). (A) Relative expression levels for miR-378a-3p, miR-378a-5p, miR-483-3p and miR-503-5p in RMS patients (ARMS and ERMS collectively considered) in comparison to NSM. Histograms indicate the mean value ± SD of independent samples (ARMS1-2-3-4-7-36-37 and ERMS1-2-3-4-12-21-23-27); (B) Relative fold change of miR-378a-3p, miR-378a-5p, miR-483-3p and miR-503-5p in RMS cell lines in comparison to NSM. Histograms represent the mean value ± SD of 4 different cell lines (RH4, RH30, RD and RD18). Y-axis values are expressed as log_2_FC (**, p < 0.05; **p < 0.01). (TIFF 91 KB)

Additional file 3: Table S2: Description of worksheet. Target Genes Gene Annotation = > ENSEMBL annotation for miR-378a-3p target genes. Target Genes GO Annotation = > ENSEMBL GO annotations for miR-378a-3p target genes; cancer- and muscle- associated categories are highlighted in blue. TGT Genes Reactome Enrichment = > analysis of over representation of Reactome Pathways in the miR-378a-3p target genes. TGT Genes DAVID Clustering = > most significant functional clusters from a DAVID analysis of the miR-378a-3p target genes. TGT Genes FI Network no linkers = > graphical representation and description of the Functional Interactions associated with the network edges of a FI network analysis performed on the miR-378a-3p target genes. No linker genes (functional links not present in the original gene list) were considered in this analysis. TGT Genes FI Network – Pathways = > pathway (CellMap, Reactome, KEGG, NCI Panther and BioCarta) enrichment analysis of the FI network. FI Network GO Enrichment = > Graphical representation and table of the most significant results of a Network Ontology Analysis on the FI network, keeping into account the functional links (edges) between the network nodes. FI Network GO (Cellular Component, Biological Process, Molecular Function) = > enrichment analysis, category by category, of the GO annotation associated with the FI network. TGT Genes Clustered FI Network = > cluster (module) analysis of the FI network, searching for functional modules and associated pathway over representation. Clustered FI Network + Linkers = > graphical representation and analysis of the over represented pathways associated with the network edges of a (clustered) FI network analysis performed on the miR-378a-3p target genes. Linker genes (functional links not present in the original gene list) were considered in this analysis, so as to give a broader view of the functional significance of the identified target gene set. (XLS 5 MB)

Additional file 4: Figure S2: Levels of transfected miR-378a-3p mature mimics in RMS cell lines. Relative expression of miR-378a-3p by Q-PCR at 72 h post transfection in RH30 and RD cells compared with miR-Ctr transfected cells. Levels of miR-378a-5p were measured to confirm the specificity of miR-378a-3p mimic transfection. Three independent experiments were performed. Comparing with respective miR-Ctr, **p < 0.01. (TIFF 56 KB)
