# Supplementary material for: Deep Sequencing the microRNA profile in rhabdomyosarcoma reveals down-regulation of miR-378 family members
Source: BMC Cancer. 2014 Nov 25;14:880. doi: 10.1186/1471-2407-14-880 (PMC4289215; doi:10.1186/1471-2407-14-880)
Supplement: Supplementary file 1 — Additional file 1: Table S1: Expression of miR-378 family members in RMS tumours. (A) miR-378 molecules differentially expressed in ARMs vs. NMS, with NMS baseline. (B): miR-378 molecules differentially expressed in ERMS vs. NMS, with NMS baseline. The members of the miR-378 family have FC values always negative and significant in both comparisons, indicating a strong under-expression in the investigated ARMS and ERMS samples. (DOC 43 KB) [file 12885_2014_5127_MOESM1_ESM.doc]

| **A** |  |  |  |  |
| --- | --- | --- | --- | --- |
| **miRNA_ID** | **log_2_FC** | **log_2_CPM** | **p-value** | **FDR** |
| hsa-miR-378a-5p | -6.97 | 11.64 | 0.00000 | 0.00000 |
| hsa-miR-378a-3p | -6.57 | 13.79 | 0.00000 | 0.00000 |
| hsa-miR-378f | -6.51 | 7.01 | 0.00000 | 0.00000 |
| hsa-miR-378c | -6.33 | 12.36 | 0.00000 | 0.00001 |
| hsa-miR-378h | -6.28 | 4.35 | 0.00000 | 0.00000 |
| hsa-miR-378i | -5.79 | 6.45 | 0.00000 | 0.00001 |
| hsa-miR-378d | -5.46 | 5.52 | 0.00000 | 0.00003 |
| hsa-miR-378e | -5.44 | 9.30 | 0.00000 | 0.00012 |
| hsa-miR-378b | -4.53 | 4.42 | 0.00000 | 0.00006 |
| hsa-miR-378g | -4.82 | 4.55 | 0.00001 | 0.00048 |
| hsa-miR-378j |  |  |  |  |
|  |  |  |  |  |
| **B** |  |  |  |  |
| **miRNA_ID** | **log_2_FC** | **log_2_CPM** | **p-value** | **FDR** |
| hsa-miR-378a-5p | -5.04 | 11.64 | 0.00002 | 0.00202 |
| hsa-miR-378a-3p | -4.60 | 13.79 | 0.00005 | 0.00351 |
| hsa-miR-378f | -4.47 | 7.01 | 0.00005 | 0.00351 |
| hsa-miR-378c | -4.17 | 12.36 | 0.00026 | 0.01105 |
| hsa-miR-378h | -4.14 | 4.35 | 0.00002 | 0.00207 |
| hsa-miR-378i | -4.05 | 6.45 | 0.00013 | 0.00773 |
| hsa-miR-378d | -3.96 | 8.81 | 0.00030 | 0.01160 |
| hsa-miR-378b | -3.52 | 5.52 | 0.00098 | 0.03343 |
| hsa-miR-378e | -3.48 | 9.30 | 0.00172 | 0.04445 |
| hsa-miR-378g | -3.43 | 4.55 | 0.00129 | 0.03831 |
| hsa-miR-378j |  |  |  |  |
|  |  |  |  |  |
