# Supplementary figures and images for: Deep Sequencing the microRNA profile in rhabdomyosarcoma reveals down-regulation of miR-378 family members
Source: BMC Cancer. 2014 Nov 25;14:880. doi: 10.1186/1471-2407-14-880 (PMC4289215; doi:10.1186/1471-2407-14-880)

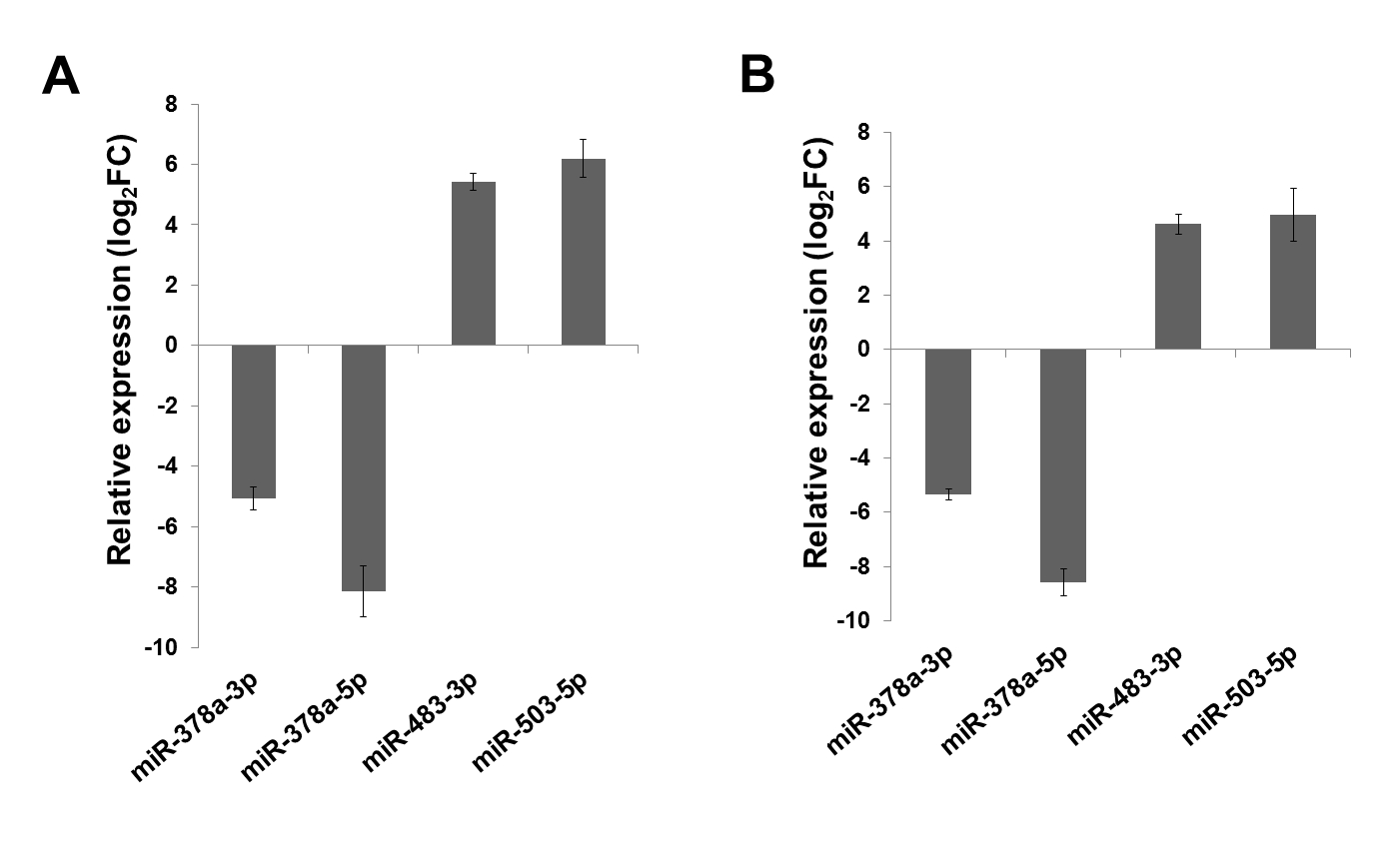

Supplement: Supplementary file 2 — Additional file 2: Figure S1: Validation of miRNA array data by quantitative real-time polymerase chain reaction (Q-PCR). (A) Relative expression levels for miR-378a-3p, miR-378a-5p, miR-483-3p and miR-503-5p in RMS patients (ARMS and ERMS collectively considered) in comparison to NSM. Histograms indicate the mean value ± SD of independent samples (ARMS1-2-3-4-7-36-37 and ERMS1-2-3-4-12-21-23-27); (B) Relative fold change of miR-378a-3p, miR-378a-5p, miR-483-3p and miR-503-5p in RMS cell lines in comparison to NSM. Histograms represent the mean value ± SD of 4 different cell lines (RH4, RH30, RD and RD18). Y-axis values are expressed as log2FC (**, p < 0.05; **p < 0.01). (TIFF 91 KB) [file 12885_2014_5127_MOESM2_ESM.tiff]

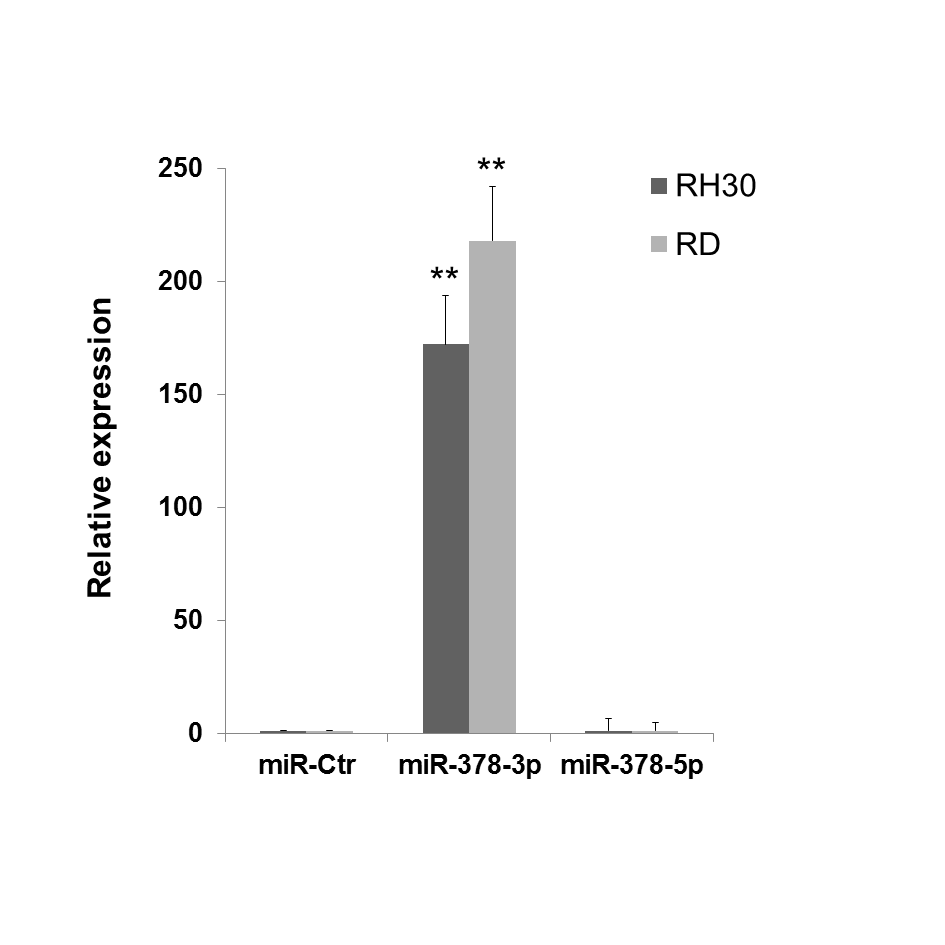

Supplement: Supplementary file 4 — Additional file 4: Figure S2: Levels of transfected miR-378a-3p mature mimics in RMS cell lines. Relative expression of miR-378a-3p by Q-PCR at 72 h post transfection in RH30 and RD cells compared with miR-Ctr transfected cells. Levels of miR-378a-5p were measured to confirm the specificity of miR-378a-3p mimic transfection. Three independent experiments were performed. Comparing with respective miR-Ctr, **p < 0.01. (TIFF 56 KB) [file 12885_2014_5127_MOESM4_ESM.tiff]
